# The Antidepressant Duloxetine Inhibits Platelet Function and Protects against Thrombosis

**DOI:** 10.3390/ijms23052587

**Published:** 2022-02-26

**Authors:** Patricia A. Lozano, Ahmed B. Alarabi, Sarah E. Garcia, Erica T. Boakye, Hendreta T. Kingbong, Elie Naddour, Daniel Villalobos-García, Precious Badejo, Medhat S. El-Halawany, Fadi T. Khasawneh, Fatima Z. Alshbool

**Affiliations:** 1 Department of Pharmacy Practice, Irma Lerma Rangel College of Pharmacy, Texas A&M University, Kingsville, TX 78363, USA; patricialozano@tamu.edu (P.A.L.); alarabi@tamu.edu (A.B.A.); 2 School of Pharmacy, The University of Texas at El Paso, El Paso, TX 79902, USA; segarcia@minres.utep.edu (S.E.G.); etboakye@miners.utep.edu (E.T.B.); htkingbong@miners.utep.edu (H.T.K.); enaddour@miners.utep.edu (E.N.); 3 Department of Pharmaceutical Sciences, Irma Lerma Rangel College of Pharmacy, Texas A&M University, Kingsville, TX 78363, USA; danielvg@tamu.edu (D.V.-G.); preciousbadejo@tamu.edu (P.B.); m.elhalawany@exchange.tamu.edu (M.S.E.-H.)

**Keywords:** platelet, thrombosis, hemostasis, MDD, CVD, drug repurposing

## Abstract

While cardiovascular disease (CVD) is the leading cause of death, major depressive disorder (MDD) is the primary cause of disability, affecting more than 300 million people worldwide. Interestingly, there is evidence that CVD is more prevalent in people with MDD. It is well established that neurotransmitters, namely serotonin and norepinephrine, are involved in the biochemical mechanisms of MDD, and consequently, drugs targeting serotonin-norepinephrine reuptake, such as duloxetine, are commonly prescribed for MDD. In this connection, serotonin and norepinephrine are also known to play critical roles in primary hemostasis. Based on these considerations, we investigated if duloxetine can be repurposed as an antiplatelet medication. Our results-using human and/or mouse platelets show that duloxetine dose-dependently inhibited agonist-induced platelet aggregation, compared to the vehicle control. Furthermore, it also blocked agonist-induced dense and α-granule secretion, integrin αIIbβ3 activation, phosphatidylserine expression, and clot retraction. Moreover duloxetine-treated mice had a significantly prolonged occlusion time. Finally, duloxetine was also found to impair hemostasis. Collectively, our data indicate that the antidepressant duloxetine, which is a serotonin-norepinephrine antagonist, exerts antiplatelet and thromboprotective effects and inhibits hemostasis. Consequently, duloxetine, or a rationally designed derivative, presents potential benefits in the context of CVD, including that associated with MDD.

## 1. Introduction

While cardiovascular disease (CVD) is the leading cause of death worldwide [1], major depressive disorder (MDD), also known as depression, is one of the most common mental disorders in the United States [2]. Interestingly, clinical evidence estimated that MDD is present in one out of five patients with CVD, including coronary heart disease, and an imbalance of serotonergic mechanisms (serotonin regulation) is involved in the pathogenesis of both diseases. Thus, while serotonin is an important neurotransmitter in the central nervous system [3] and its deficiency is a key feature of MDD, a number of CVDs have been linked to elevated plasma levels of serotonin [4]. To this end, serotonin is secreted by blood platelets [5] and plays diverse roles in the cardiovascular system, including potentiation of platelet aggregation [6]. Thus, serotonin could potentially serve as a drug target to both disease states.

Platelets are anucleated cells that play a critical role in hemostasis. Although platelet activation is required in response to blood vessel injury to arrest bleeding, it is also responsible for the development of thrombotic events [7,8]. Platelets are activated by several agonists, such as thrombin and ADP, that bind to specific platelet membrane receptors. Upon activation, platelets release a host of molecules from their granules, including serotonin (5-hydroxytrytamine, 5-HT), which is released from the dense granules [9]. It is considered as a weak platelet agonist, but it has the ability to amplify the aggregatory response [10]. As for the uptake of serotonin from the blood plasma by platelets, it is dependent on the serotonin transporter (SERT) [5], with the sequestration into the dense granules via the vesicular monoamine transporter (VMAT1). The elevated level of blood plasma 5-HT level also activates a specific platelet surface-localized 5-HT receptor (5-HT2AR) [11].

With regard to drugs that modulate serotonin, in general, the selective serotonin reuptake inhibitors (SSRIs) are the first antidepressant drugs that exert a significant blockade on SERTs not only in the central nervous system, but also in the blood platelets. Thus, SSRI treatment may be associated with impaired platelets, leading to an increased risk of gastrointestinal hemorrhage, as well as intraoperative and postoperative blood loss [12]. Another class of medications that is also widely prescribed for the treatment of MDD is the serotonin-norepinephrine reuptake inhibitors (SNRIs), which are thought to be associated with a better response and a more rapid onset of activity in comparison to SSRIs. These agents could also potentially exert antiplatelet effects. Nonetheless, despite the important, and probably underestimated role of serotonin in platelet function, there are no SERT antagonists approved by the Food and Drug Administration (FDA) for use as antiplatelet therapies [13].

It is known that new drug discovery and development is a very lengthy and costly process [14]. For this reason, the repurposing of existing drugs that have already been approved by the FDA offers clear advantages. Duloxetine (Cymbalta) is the first generation SNRI that received FDA approval in 2004, with a considerable amount of evidence in support of its efficacy, safety, and tolerability. Aside from MDD, duloxetine has been prescribed for other psychiatric disorders, and several clinical conditions such as urinary incontinence, neuropathic pain, and fibromyalgia [15]. Taking the aforementioned issues into consideration, in the present study we aim to investigate the antiplatelet effects of duloxetine for potential drug repurposing. Our results demonstrate that duloxetine inhibits agonist-induced platelet aggregation, dense and α-granule secretion, GPIIb-IIIa activation, phosphatidylserine (PS) exposure, as well as clot retraction. Furthermore, duloxetine delayed both the occlusion start and occlusion times in an in vitro model of hemostasis. Finally, duloxetine was found to increase the occlusion time and the tail bleeding time of treated mice These findings underscore the potential of duloxetine, an existing drug, for repurposing as an antiplatelet agent, especially in CVD patients with MDD. This should positively impact patient adherence and/or reduce the risk of developing side effects.

## 2. Results

### 2.1. Duloxetine Inhibits Agonist-Induced In Vitro and Ex Vivo Platelet Aggregation in a Dose-Dependent Manner

**Figure 1:**
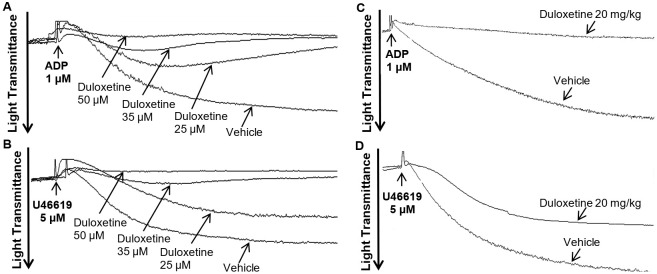
Duloxetine inhibits agonist-induced in vitro (**A**,**B**) and ex vivo (**C**,**D**) platelet aggregation. Human platelets were pre-incubated with increasing doses of duloxetine (25, 35 and 50 µM) or the vehicle for one minute (**A**,**B**), or mice were injected with 20 mg/kg duloxetine one hour prior to platelet collection (**C**,**D**). Platelets were stimulated with ADP (1 µM; (**A**,**C**)) or U46619 (5 µM; (**B**,**D**)), before the aggregation response was measured using an aggregometer. These experiments were repeated four times using blood obtained from three different healthy human subjects, or pooled from six-eight mice each time, as applicable.

To determine whether duloxetine has an impact on platelet activity, we first investigated its effect on human platelet aggregation, under in vitro settings. It was found that 25 μM of duloxetine significantly inhibited ADP-induced platelet aggregation (1 µM; Figure 1A; 48% ± 8.0), in comparison with the vehicle-treated platelets. This inhibition was found to be dose-dependent (Figure 1A; 73% ± 9.0 at 35 µM, 88% ± 7.0 at 50 µM). Comparable results were observed with the agonist U46619 (5 µM; Figure 1B; 36% ± 10 at 25 µM, 70% ± 8.0 at 35 µM, 84% ± 9.0 at 50 µM). Of note, based on these data and the fact that 35 µM of duloxetine has the capacity to exert significant inhibitory effects on platelet function regardless of the platelet function assay and/or agonist used, this dose was selected for further in vitro experimentation. Following this, we examined its ex vivo effects by injecting mice with 20 mg/kg of duloxetine, which was selected based on a literature review [16,17,18,19,20,21], before platelets were collected and their aggregation response examined. Indeed, duloxetine significantly reduced ADP- (58% ± 13), and U46619 (43% ± 11)-induced platelet aggregation (Figure 1C,D, respectively).

### 2.2. Duloxetine Inhibits P-Selectin Expression and ATP Release in Stimulated Platelets

**Figure 2:**
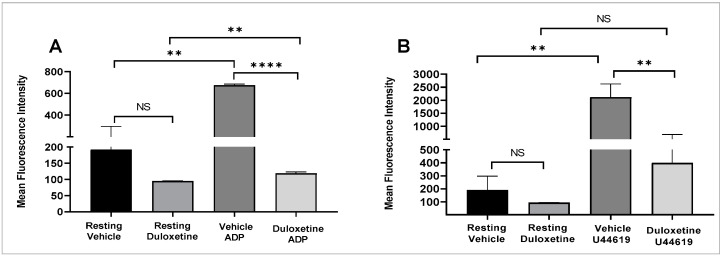
Duloxetine inhibits platelet α-granule secretion. Human platelets were pre-incubated with duloxetine (35 µM) or the vehicle for one minute. Platelets were stimulated with ADP (1 µM; (**A**)) or U46619 (5 µM; (**B**)) and incubated with FITC–conjugated P-selectin antibody. Fluorescent intensities were measured by flow cytometry. Average mean fluorescence intensities shown (** *p* < 0.01; **** *p* < 0.0001; NS, nonsignificant). These experiments were repeated three times, using blood obtained from three different healthy human subjects. The results were compared by running a one-way ANOVA followed by Tukey’s multiple comparisons test using GraphPad Prism.

P-selectin translocates from α-granules to the platelet surface [22] to amplify initial platelet activation [23]. Given the important role this process plays in platelet function and thrombus formation, we investigated the effects of duloxetine (35 µM) on P-selectin surface levels using flow cytometry. It was observed that duloxetine-treated platelets exhibited significantly lower surface expression of P-selectin, when stimulated by 1 µM ADP or 5 µM U46619 (Figure 2A,B, respectively). Interestingly, duloxetine appeared to lower the basal levels of P-selectin, but these effects did not reach statistical significance.

**Figure 3:**
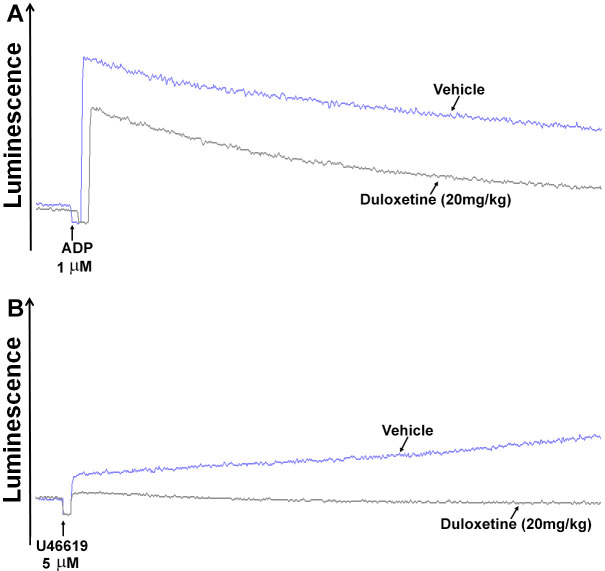
Duloxetine inhibits dense granule secretion ex vivo. Mice were injected with 20 mg/kg of duloxetine one hour prior to platelet collection. Platelets were incubated with 12.5 μL of luciferase luciferin before stimulation with either ADP (1 µM; (**A**)) or U46619 (5 µM; (**B**)) for 5 min. ATP dense granules release (luminescence) was measured using an aggregometer. These experiments were repeated three times, using blood pooled from six-eight mice each time.

We next investigated the effect of duloxetine under ex vivo experimental conditions in the context of dense-granule secretion. Our findings revealed that ATP secretion was inhibited in platelets obtained from mice that were injected with 20 mg/kg duloxetine, but not the vehicle control ((Figure 3A, 42% ± 8.0) and (B, 47% ± 12.0)).

### 2.3. Duloxetine Inhibits Agonist-Induced Glycoprotein IIb-IIIa Activation and Phosphatidylserine Expression in Stimulated Platelets

**Figure 4:**
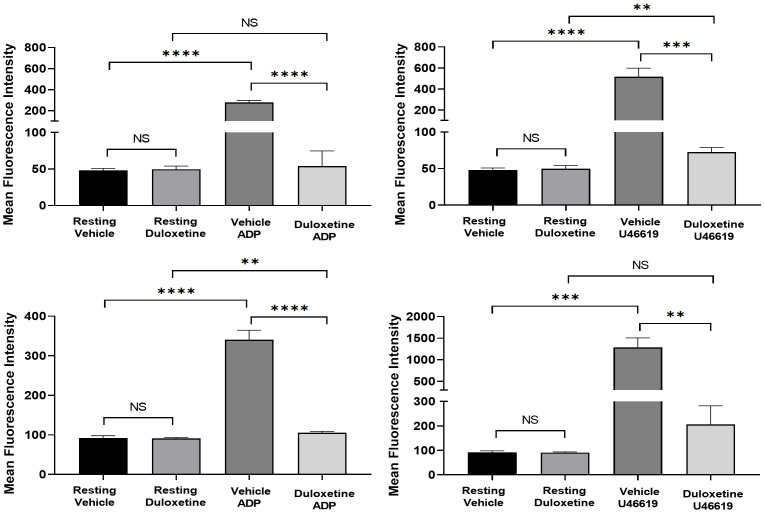
Duloxetine inhibits glycoprotein IIb-IIIa activation (**A**,**B**) and phosphatidylserine expression (**C**,**D**). Washed human platelets were pre-incubated with duloxetine (35 µM) or the vehicle for one minute. Platelets were incubated with FITC-conjugated PAC-1 antibody (**A**,**B**) and Annexin V antibody (**C**,**D**) before stimulation with ADP (1 µM; (**A**,**C**)) or U46619 (5 µM; (**B**,**D**)). Fluorescent intensities were measured by flow cytometry. Average mean fluorescence intensities shown (** *p* < 0.01; *** *p* < 0.001; **** *p* < 0.0001; NS, nonsignificant). These experiments were repeated three times, using blood obtained from three different healthy human subjects. The results were compared by running a one-way ANOVA followed by Tukey’s multiple comparisons test using GraphPad Prism.

Activation of integrin glycoprotein IIb-IIIa (GPIIb-IIIa, also known as αIIbβ3) is required for platelet aggregation [24]. Moreover, phosphatidylserine (PS) is important for coagulation, were activated platelets express PS on their surface [25]. To assess whether duloxetine inhibits GPIIb-IIIa activation and modulates PS exposure, we measured the surface levels (on human platelets) of activated GPIIB-IIIa and PS by flow cytometry. Indeed, we found that ADP (1 µM; Figure 4A,C) and U46619 (5 µM; Figure 4B,D) induced activation of the GPIIB-IIIa integrin and PS expression were significantly inhibited (reduced) in duloxetine-treated platelets, compared with the vehicle-treated platelets. These data are consistent with duloxetine’s effects on aggregation and secretion. Taken together, these results indicate that duloxetine impairs platelet aggregation, secretion, integrin activation, as well as PS exposure.

### 2.4. Duloxetine Impairs Clot Retraction

**Figure 5:**
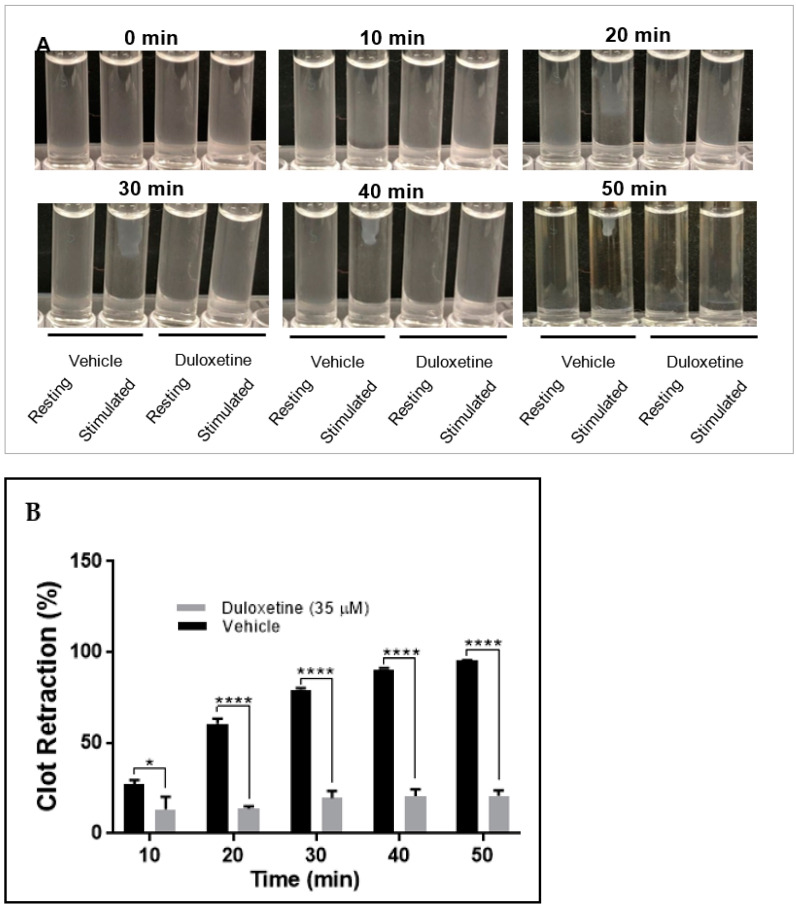
Duloxetine blocks clot retraction. Washed human platelets were isolated and incubated with either duloxetine (35 µM) or the vehicle for 1 min and transferred to a glass cuvettes. Clot retraction was initiated by adding thrombin (0.1 U/mL), and monitored over time, with images taken via a digital camera every 10 min (**A**). Data quantification is shown in panel (**B**). This experiment was repeated three times, using blood obtained from three different healthy human subjects. The results were compared by running the student *t*-test using GraphPad Prism (* *p* < 0.05; **** *p* < 0.0001).

Clot retraction is a complex process mediated by an internal signaling cascade, where the activation of integrin GPIIb-IIIa is required [24]. We investigated whether duloxetine inhibits clot retraction. Our results showed that duloxetine (35 µM) blocked clot retraction, when compared to the control (Figure 5). These data indicate that duloxetine inhibits GPIIb-IIIa outside-in signaling.

### 2.5. Duloxetine Prolongs Occlusion Time and Tail Bleeding Time

**Figure 6:**
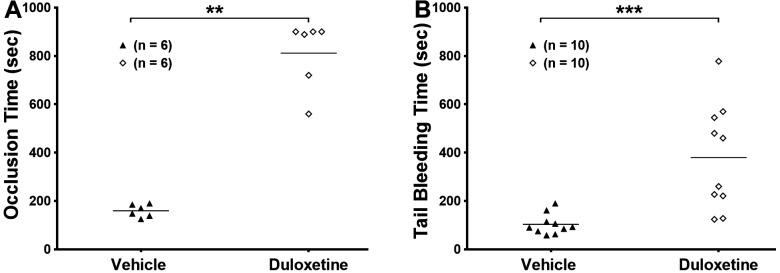
Duloxetine prolongs the occlusion and tail bleeding time. Mice were IV-injected with 20 mg/kg of duloxetine or the vehicle, and after hour they were subjected to the ferric chloride-induced thrombosis model (**A**) or bleeding time assay (**B**). Each point represents the occlusion time of a single animal, or the tail bleeding of a single mouse (**B**), respectively. Data were compared by running the Mann—Whitney test using GraphPad Prism (** *p* < 0.01; *** *p* < 0.001).

Abnormal-platelet hyperactivity is also known to promote thrombogenesis [26], which can lead to CVDs such as heart attacks and strokes [27]. Thus, we tested whether duloxetine would also inhibit thrombus formation. Our data show that mice that received a tail IV injection of duloxetine (20 mg/kg) and were subjected to the FeCl3 carotid artery injury thrombosis model [28] exhibited significantly prolonged occlusion times, compared with vehicle-injected mice (Figure 6A; *n* = 6 each; *p* = 0.0022). We next evaluated the effect of duloxetine on the hemostasis response, by measuring tail bleeding time. It was found that the bleeding time of duloxetine-injected mice was much longer than that of vehicle-treated mice (Figure 6B; *n* = 10 each; *p* = 0.0001). Together, these results demonstrate that duloxetine decreases the risk of thrombotic events, albeit it also interferes with physiological hemostasis.

### 2.6. Duloxetine Modulates Hemostasis Response in Human Platelets

**Figure 7:**
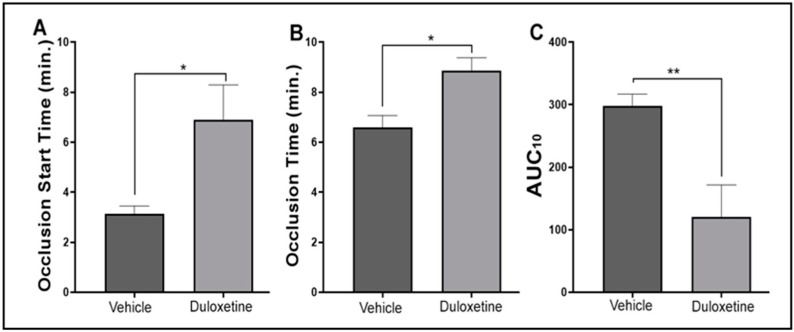
Duloxetine modulates hemostasis in human platelets. Blood was collected from healthy human subjects, treated with 50 µM duloxetine or the vehicle for one minute before being subjected to T-TAS01 system analysis (PL chip flow under arterial shear stress conditions on a collagen coated surface). The occlusion start time (**A**), occlusion time (**B**) and area under the curve/AUC_10_ (**C**) were then measured. These data were obtained from six different healthy human subjects. Data were compared by running the student *t*-test using GraphPad Prism (* *p* < 0.05; ** *p* < 0.01).

Finally, we sought to investigate whether duloxetine exerts any effects on hemostasis in humans. This was achieved by employing the T-TAS 01 System with PL chip that uses arterial shear flow to analyze platelet-rich thrombus formation (primary hemostasis) of whole blood on a collagen-coated surface [29,30]. Our results showed that duloxetine prolonged the occlusion time and the occlusion start time as well as reduced the area under the curve (AUC) dose-dependently, indicating inhibition of platelet activation (Figure 7).

## 3. Discussion

There is an increasing amount of evidence and observational studies documenting that major depression and heart disease are likely to occur simultaneously, in the same individual, albeit the mechanisms underlying this comorbidity are not fully elucidated [31]. These two disease states are persistent, frequently under-recognized and can lead to death [32]. Interestingly, it was previously shown in human subjects, that increased platelet activation and aggregation is a feature of MDD [22]. Furthermore, the study also suggested that the resolution of depression using an antidepressant medication reduces platelet activation [22]. In this connection, it was found that the SERT molecules that are expressed by platelets exhibit pharmacological properties that are very comparable to those of the CNS. This similarity has been accepted in the literature, including in terms of sensitivity to antidepressants; thus, the SERT state in platelets mirrors that in serotonergic neurons in CNS [23,24,25,26]. Moreover, mutations in SERT were found to exert CNS and platelet consequences [5,27,28].

Platelets function as exocytotic cells, thereby they have the capacity to secrete a host of effector molecules that mediate hemostasis, thrombosis, and inflammatory responses [29]. Current FDA-approved antiplatelet therapies are associated with a host of adverse drug reactions. Moreover, the side effects of some of these agents are amplified in patients with comorbidities, namely CVD and another disease state. Hence, there is significant interest in pursuing new pharmacological agents that not only have a better safety profile, but also could potentially be effective in managing these comorbid diseases (multiple disorders). To this end, given the significant amount of time and cost needed to get a drug approved by the FDA [30], and the fact that many drug candidates never make it through the approval process [33], drug repurposing or rediscovery presents an attractive alternative approach [34,35,36]. In this approach, FDA-approved drugs are investigated for a new therapeutic activity, thereby offering several advantages such as well-defined pharmacological and safety profiles that have potentially been gathered through years of clinical use [34,35,36].

Based on the aforementioned considerations, the goal of the present study is to employ the concept of drug rediscovery in order to identify an agent that might be effective in the context of MDD and CVD pathophysiological conditions [37]. Thus, we sought to repurpose the well-tolerated antidepressant duloxetine as an antiplatelet agent, as was done with separate FDA-approved agents [38,39,40]. To achieve our goal, we performed a series of assays using human platelets as well as live mice and their platelets. Our results revealed a marked decrease in agonist-triggered aggregation, degranulation, and GPIIb-IIIa activation by duloxetine, albeit the doses may not be therapeutically relevant. Of note, duloxetine appeared to lower the basal levels of P-selectin, although this did not reach statistical significance, which suggests that it may be exerting some agonist-independent effects on the α granules and/or their storage capacity. Interestingly, no apparent effects were observed in the context of the basal levels of GPIIb-IIIa and PS expression. We also observed similar inhibition of some of these platelet functional responses when duloxetine was injected into mice before their platelets were isolated for experimentation. In support of our data, a previous report showed that fibrinogen enhances SERT activity in platelets and that GPIIb-IIIa can in fact directly interact with the C terminus of SERT [41]. It was also observed that duloxetine inhibited PS exposure and clot retraction. Interestingly, knockout mice lacking integrin β3 displayed diminished platelet SERT activity [41].

Our in vitro and ex vivo data thus far provide evidence that duloxetine could have thromboprotective properties. In order to address this issue, we examined whether duloxetine modulates thrombogenesis and hemostasis in live mice. We used a dose (20 mg/kg) that corresponds to the therapeutic range for MDD treatment in humans [17,42]. It was observed that duloxetine-treated mice present a prolonged occlusion time in comparison with the vehicle-treated mice. Furthermore, it also significantly extended the tail bleeding time. Hence, these findings are not only in accordance with the duloxetine’s observed effects on platelet function, but also with the recommendation to stop duloxetine therapy a few days before surgery [15], in part, to avoid bleeding complications. Further support of this notion derives from additional data we obtained using human blood, in which platelet activity was analyzed using the T-TAS 01 PL chip system. This system, which allowed us to explore the effect of duloxetine on platelets under physiologically relevant conditions/primary hemostasis [43], did show that duloxetine modulates hemostasis, albeit its dose may not be therapeutically relevant. This issue should be considered in patients who are under high risk of bleeding, those who will undergo a surgical procedure, and/or those who are prescribed or need to be prescribed antithrombotic agents.

Further studies are warranted if duloxetine is to be repurposed (to assess its clinical utility) as an antiplatelet therapy, and whether it would offer a great advantage for MDD patients with CVD. Furthermore, drug-drug interactions must be carefully considered by clinicians when MDD and CVD patients are prescribed with more than one medication to determine the safe dosage range in short- and long-term treatment protocols. Of note, duloxetine is also used to treat different types of chronic pain, but more studies are still needed to determine the dosage range and its long-term efficacy and safety in this area [15]. Moreover, while platelets express the serotonin receptor 5-HTA2R, its inhibitors have produced mild and/or contradictory effects in rodent, dog, and human studies. Finally, we cannot exclude the possibility that the doses we selected may exert toxicity, as they were selected based on a thorough dose-response analysis, with those found to exert significant inhibitory effects selected for further experimentation. While this may be the case, we did observe that duloxetine’s inhibitory effects were dose-dependent, which may support some level of specificity, a notion that will be investigated in future studies.

## 4. Materials and Methods

### 4.1. Reagents and Materials

(S)-Duloxetine hydrochloride, ADP, and fibrinogen were purchased from Sigma Aldrich (St. Louis, MO, USA). The agonist U46619 was purchased from Abcam (Cambridge, MA, USA). Platelet aggregometry supplies such as glass cuvettes and magnetic stir bars, and luciferin-luciferase (Chrono-Lume) were purchased from the Chrono-Log Corporation (Havertown, PA, USA). Fluorescein isothiocyanate (FITC)–conjugated anti–P-selectin, PAC1, and Annexin V were purchased from BD Biosciences (San Jose, CA, USA).

### 4.2. Human Blood Samples

Human blood studies were approved by the Institutional Review Board (IRB). Non-smoker and healthy participants were asked to read and sign a written consent form and a donor’s bill of rights form prior to blood collection.

### 4.3. Animals

C57BL/6 mice were purchased from the Jackson Laboratory (Bar Harbor, ME, USA). All animals used for experiments were males and 10–14 weeks of age. Mice were housed with access to water and food in the vivarium facility with controlled temperature (24 °C), oxygen and a 12-h light/dark cycle. All experiments involving animals were performed in compliance with the institutional guidelines and were approved by the Institutional Animal Care and Use Committee (IACUC).

### 4.4. Human and Mouse Platelet-Rich Plasma Preparation

Blood was drawn from healthy participants, whereas mouse blood was collected directly from the heart under anesthesia. Human and mouse blood samples were collected in tubes containing 3.8% *w/v* sodium citrate (1 part of the coagulation solution to 9 parts of blood). Human and mouse platelet-rich plasma (PRP) was obtained by centrifugation at room temperature. Platelet count was obtained using an automated hematology analyzer and adjusted to 7 × 10^7^ platelet/mL for each experiment.

### 4.5. Washed Platelets Preparation

Washed human platelets were obtained as described previously [44,45,46,47]. PRP was isolated and centrifuged at 150× *g* for 10 min at 20 °C in the presence of 0.37 U/mL apyrase and 10 ng/mL PGI_2_. PRP was separated and centrifuged at 900× *g* for 10 min. Pelleted containing-platelets were resuspended in HEPES/Tyrode’s buffer (containing 1 mM EGTA, 0.37 U/mL apyrase, and 10 ng/mL PGI_2_). Platelets were washed and resuspended in HEPES/Tyrode’s buffer (pH 7.4) deprived of EGTA, apyrase, or PGI_2_. The final platelet count was adjusted to 4 × 10^8^ platelets/mL, unless otherwise indicated.

### 4.6. In Vitro Platelet Aggregation

Human platelet-rich plasma was incubated with either duloxetine (25, 35 and 50 µM) or the vehicle control (DMSO) for one minute at room temperature prior to experiments. Platelets (250 μL; 7 × 10^7^/mL) were added into glass cuvettes with stir bars followed by the addition of the agonists ADP (1 µM) and U46619 (5 µM). Platelet aggregation was measured by the turbidometric method using the model 700 aggregometer system (Chrono-Log Corporation, Havertown, PA, USA). Data were quantified at the maximum aggregation response achieved in the presence of duloxetine, and “Column Statistics” were performed using GraphPad Prism to calculate the Mean and Standard Deviation (SD).

### 4.7. Ex Vivo Platelet Aggregation and ATP Release

Mice were injected with a pharmacologically relevant dose of duloxetine (20 mg/kg) [17,48] or the vehicle (DMSO) using the tail intravenous (IV) route one hour prior to blood collection. Platelets were placed into siliconized cuvettes and stimulated with ADP (1 µM) and U46619 (5 µM). Platelet aggregation was measured as described before. For ATP/dense granules release, the luciferase substrate/luciferase mixture (12.5 μL, Chrono-Log) was added, before agonist stimulation (ADP (1 µM) and U46619 (5 µM)). Data were quantified at the maximum aggregation and secretion responses achieved in the presence of duloxetine, and “Column Statistics” were performed using GraphPad Prism to calculate the Mean and Standard Deviation (SD).

### 4.8. Flow Cytometric Analysis

Flow cytometry assay was conducted as previously described [49]. Human washed platelets were incubated with either 35 µM duloxetine or the vehicle for 1 min at room temperature. Platelet stimulation was initiated by adding ADP (1 µM) and U46619 (5 µM) for 5 min, before they were fixed with 10% formaldehyde for 30 min at room temperature. After fixation, platelets were incubated with FITC–conjugated CD62P (P-selectin), PAC-1, or Annexin V antibodies at room temperature for 30 min in the dark. Platelets were diluted (2.5 fold) with HEPES/Tyrode buffer (pH7.4) and transferred to FACS tubes. Finally, fluorescent intensities were measured using a BD Accuri C6 Flow Cytometer and results were analyzed using CFlow Plus (BD Biosciences). Each experiment was repeated three times, using blood from three different donors. Data were compared by running a one-way ANOVA followed by Tukey’s multiple comparisons test.

### 4.9. Fibrin Clot Retraction Assay

Fibrin clot retraction assay was performed as previously described [45,46,47,50]. Human washed platelets (500 μL aliquot) were resuspended at 1 × 10^8^/mL HEPES-Tyrode buffer (pH = 7.4), before CaCl_2_ was added (final concentration 1 mM). Next, duloxetine (35 μg) or the vehicle (DMSO) was added, they were then incubated for one minute prior to the initiation of clot retraction, by adding fibrinogen (500 μg/mL) and thrombin (0.1 U/mL). Using a digital camera, pictures were taken at time intervals of 10 min up to 50 min. The clot retraction data were quantified using the ImageJ software, by analyzing the volume of the clot in both the control and treated platelets, and the results were compared by running the student *t*test using GraphPad Prism.

### 4.10. Tail Bleeding Time

Hemostasis was examined by performing the tail transection technique as described previously [45,46,49]. Mice were tail IV-injected with duloxetine (20 mg/kg) or the vehicle for an hour. Then mice were anesthetized using isoflurane and properly placed on a homoeothermic blanket (37 °C). After the tip of tail was transected (5 mm segment) using a sterile scalpel, the tail was placed in saline solution at 37 °C and the time until bleeding stoppage was recorded and compared by running the Mann—Whitney test using GraphPad Prism. This time was not considered complete until bleeding had stopped for 60 s. Bleeding time did not continue beyond 10 min for statistical analysis purposes and to reduce blood loss from mice.

### 4.11. Human Hemostasis Assessment

Human primary hemostasis was analyzed using a T-TAS 01 instrument (Fujimori Kogyo Co., Ltd. (ZACROS), Tokyo, Japan) by employing the PL-chip. BAPA (DiaPharma Group Inc., West Chester, OH, USA) anticoagulated whole blood samples from healthy human donors were incubated with the vehicle or 50 µM duloxetine for 1 min, before the blood was subjected to flow through the PL chip collagen coated channels under a share rate of 1500 s^−1^, according to the manufacturer’s instructions. Measurements of the T-TAS 01 parameters, including occlusion start time (OST), occlusion time (OT; within 10 min or when a pressure reading reaches 60 kPa), and the area under the pressure curve (AUC), were taken in duplicates. Data were compared by running the student *t*-test using GraphPad Prism.

### 4.12. In Vivo Ferric Chloride Carotid Artery Injury–Induced Thrombosis Model

In vivo thrombosis model assay was performed in the same manner as described previously [45,46,47,49,51]. Mice received a tail IV injection of duloxetine (20 mg/kg) or the vehicle prior to the procedure. Animals were deeply anesthetized with avertin (2.5%) and placed on a homoeothermic blanket (37 °C). The left carotid artery was exposed and cleaned with saline solution. The baseline carotid artery blood flow was measured with the Transonic Micro-Flow Probe (Transonic Systems Inc., Ithaca, NY, USA). Once blood flow reached stabilization, 7.5% ferric chloride (FeCl_3_) was applied to filter paper (1-mm diameter) and placed on top of the carotid artery for three minutes. Blood flow was continuously monitored until it stopped completely (stable occlusion). Data were recorded as the time to vessel occlusion and calculated as the difference in time between stable occlusion and removal of the filter paper containing ferric chloride. Data were compared by running the Mann—Whitney test using GraphPad Prism.

### 4.13. Statistical Analysis

Data analysis was achieved using the statistical software GraphPad Prism (San Diego, CA, USA). Results were presented as mean ± SEM or SD. Statistical significance was accepted as *p <* 0.05.

## 5. Conclusions

In summary, employing a host of platelet function tests, human and mouse platelets, and in vivo animal models, our study revealed that duloxetine, which is a widely prescribed antidepressant agent, exhibits antiplatelet and antithrombotic activity, and it, or a rationally-designed derivative, has the potential to be repurposed for therapeutic purposes.

## Data Availability

Data will be made available by the corresponding authors upon request.
